# Evaluation of Cross-Protective Responses against Mayaro Virus among Chikungunya Virus-Infected Patients from Paraguay

**DOI:** 10.4269/ajtmh.25-0624

**Published:** 2026-05-05

**Authors:** Gabrielle N. Kostecki, Nicolás Aguayo, Fulvia V. Campuzano, Julia S. Ampuero, Patricia V. Aguilar

**Affiliations:** ^1^ Galveston National Laboratory, University of Texas Medical Branch, Galveston, Texas, USA;; ^2^ Department of Pathology, University of Texas Medical Branch, Galveston, Texas, USA;; ^3^ Rayos de Sol NGO, Asuncion, Paraguay;; ^4^ US Naval Medical Research Unit South, Lima, Peru;; ^5^ Center for Tropical Diseases, University of Texas Medical Branch, Galveston, Texas, USA

## Abstract

Mayaro virus (MAYV) is a mosquito-borne alphavirus endemic to Latin America and the Caribbean. It is closely related to chikungunya virus (CHIKV), which has a more global circulation that overlaps with MAYV-endemic areas. Both viruses cause similar symptoms of acute febrile illness and chronic joint pain. Human studies have attempted to determine whether CHIKV can generate cross-protective immunity against MAYV because of their close genetic and antigenic relationship but could not definitively rule out past exposure to MAYV. We investigated cross-reactive MAYV responses using paired blood samples from 15 CHIKV-infected patients from Paraguay, where there have been no reported Mayaro fever cases. These samples were collected during the acute phase of the illness and 2 to 4 weeks after the onset of symptoms. Acute-phase serum samples were confirmed positive for the presence of CHIKV viral RNA with reverse transcription polymerase chain reaction. Plaque reduction neutralization tests were performed on the samples to calculate 80% plaque reduction neutralization titers for CHIKV and MAYV. Previous MAYV exposure was detected in three CHIKV patients, suggesting that past exposure to MAYV might not be sufficient to protect against CHIKV infection. Of the other CHIKV patients without prior MAYV immunity, only one third developed low MAYV cross-neutralizing antibody responses, indicating nonreciprocity in CHIKV and MAYV cross-protection. This study provides evidence of a potential silent circulation of MAYV in Paraguay, which requires further investigation. These findings have critical implications for areas coendemic for MAYV and CHIKV and provide important advances to better understand cross-protection among alphaviruses.

## INTRODUCTION

Mayaro virus (MAYV) and chikungunya virus (CHIKV) are mosquito-borne viruses that belong to the family *Togaviridae* and genus *Alphavirus*. They cause similar clinical presentations, including fever, headache, rash, joint inflammation, myalgia, and a nearly 50% risk of severe chronic arthralgia.^1^^–^^3^ These two viruses also have a strong genetic similarity. A past analysis of domains A and B, which are the main targets of neutralizing antibodies on E2 glycoprotein, has revealed that MAYV had highly conserved similarity and identity scores of 72% and 52% to CHIKV, respectively.^4^

After its discovery in Trinidad and Tobago in the 1950s, MAYV caused outbreaks of Mayaro fever in more than 10 South American and Caribbean nations (reviewed in references 2 and 5). The virus is typically found in rural areas within the neotropics, with most outbreaks occurring in Brazil and Peru. However, human infections have also been reported in countries such as Haiti, Venezuela, Bolivia, French Guinea, Ecuador, Suriname, and Panama, and evidence of human exposure to MAYV has been documented in Mexico.^5^ Currently, MAYV’s enzootic cycle is not fully understood; it is thought that MAYV circulates between canopy-dwelling *Haemagogus* mosquito vectors and nonhuman primates in forested areas.^2^^,^^5^ However, reports of cases in Haiti, where there are no nonhuman primates, suggest a different transmission cycle than that reported in South America.^6^ Recent studies have also identified additional seropositive vertebrate hosts and potentially new vectors, like *Aedes aegypti* mosquitoes, highlighting important factors that can contribute to the risk of widespread MAYV emergence in urban and periurban areas.^7^^–^^9^

Chikungunya virus has emerged as one of the most prevalent alphaviruses in the world since first being identified in Tanzania in 1952.^10^ In contrast to MAYV, which is primarily detected only in the Americas, CHIKV has a much broader global circulation with millions of cases of illness and death reported across more than 60 countries and multiple continents worldwide.^2^^,^^3^ In 2013, CHIKV’s reach extended from sub-Saharan Africa, Asia, and the Indian Ocean Basin to the Americas and the Caribbean for the first time, causing more than 1.1 million reported cases that year.^11^^,^^12^ Chikungunya virus now persists in the Americas in both sylvatic and urban transmission cycles; urban CHIKV outbreaks most commonly infect humans through *Aedes albopictus* and *Ae. aegypti* mosquitoes.^2^^,^^3^

Because of the close antigenic and phylogenetic relationship between CHIKV and MAYV, there have been multiple efforts to determine if CHIKV immunity confers cross-protection against MAYV infection.^4^ Two animal model studies reported that pre-existing CHIKV immunity strongly cross-protected against secondary MAYV infection,^13^^,^^14^ and another showed partial cross-protection.^15^ Several other studies have shown that CHIKV vaccine candidates could generate cross-neutralizing antibodies against MAYV in mice.^16^^,^^17^ However, another CHIKV vaccine candidate, although eliciting limited MAYV-neutralizing antibody levels, was not found to confer sterilizing cross-protective immunity against heterologous MAYV infection in mice.^18^ Previous serological studies in humans indicated that natural CHIKV exposure^4^^,^^13^^,^^16^^,^^19^ as well as CHIKV vaccine-elicited immunity^16^ can generate antibodies that cross-neutralize MAYV to varying degrees *in vitro*; however, another study showed limited cross-protection.^15^ One limitation of the previous human studies is that pre-existing immunity to MAYV before CHIKV infection could not be completely ruled out without testing sera samples from both acute and convalescent (post-recovery) stages. Thus, whether CHIKV exposure generates cross-protective immunity against MAYV in humans remains to be conclusively elucidated. In this study, we investigated this question by assessing sera samples from 15 CHIKV-infected patients to explore the cross-neutralization potential of CHIKV-immune sera against MAYV.

## MATERIALS AND METHODS

**Figure 1.**
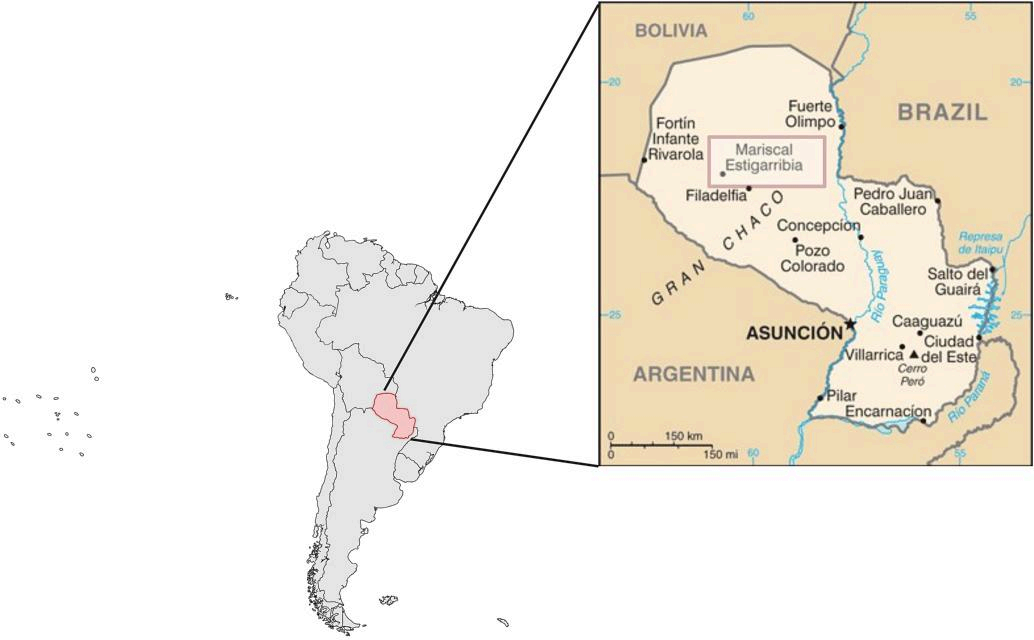
Map of Paraguay showing the location of the study site. Created by P. V. Aguilar in BioRender.

This study was part of a passive surveillance system for acute undifferentiated febrile illness in one site in Paraguay conducted by the US Naval Medical Research Unit South (NAMRU SOUTH) in collaboration with the Paraguayan Ministry of Health and the non-governmental organization (NGO) Rayos de Sol NGO. The study protocol NMRCD.2010.0010 was approved by the NAMRU SOUTH and Rayos de Sol NGO Institutional Review Boards as well as by local health authorities in Paraguay. Patients seeking medical treatment at Mariscal Estigarribia Hospital (located in the town of Mariscal Estigarribia in the Chaco Boreal region of Paraguay) (Figure 1) were recruited by health care workers if they met the following criteria: 1) 5 years of age or older; 2) temperature ≥38°C; 3) 5 days or fewer of illness; 4) clinical or laboratory evidence of a differentiated bacterial, fungal, or parasitic infection capable of causing an acute febrile illness; and 5) able to sign a consent form. Patients between 8 and 17 years old provided written assent after obtaining written consent from their parents or legal guardians. From March to June 2023, 46 febrile participants were enrolled. Acute and convalescent blood samples collected 10 to 30 days after the patients’ initial visit were obtained when possible. Acute whole-blood samples were tested on the Biofire^®^ FilmArray^®^ 2.0 System using the Global Fever Panel RUO (BioFire Defense, Murray, UT), which targets 19 pathogens, including CHIKV and dengue virus (DENV). Positive results were then confirmed by quantitative reverse transcription polymerase chain reaction using previously published protocols.^20^^,^^21^ Twenty viremic-stage (acute-stage) blood samples were confirmed to be CHIKV positive, of which four were also confirmed to be DENV positive. Of the 20 participants with confirmed CHIKV infection, 8 were females, and 12 were males, with a median age of 25 years old (range: 12–73 years old). The most common symptoms or signs in these patients were fever (100%), body aches (95%), joint pain (95%), muscle pain (90%), chills (65%), headache (65%), and malaise (35%). Two patients showed signs of bleeding or other hemorrhagic symptoms (Table 1).

**Table 1:** Demographics, clinical signs, and symptoms in chikungunya virus-infected patients in Mariscal Estigarribia, Paraguay in 2023

Patient	Age Range (years)/Sex	Sampling Date (A)	Day of Illness at Enrollment	Day of Convalescent Sample Collection	Fever	Chills	Malaise	Generalized Body Ache	Joint Pain	Muscle Pain	Headache	Rash	Hand Swelling	Medical Conditions	Film Array Results	Coinfection/Other Symptoms
1	18-24/F	March 23	4	19	Yes	Yes	No	Yes	Yes	Yes	Yes	No	No	–	CHIKV	No
2	25-34/M	March 23	3	18	Yes	Yes	No	Yes	Yes	Yes	Yes	No	No	–	CHIKV	Hemorrhagic febrile syndrome (bleeding gums)
3	35-44/M	March 23	4	20	Yes	Yes	No	Yes	Yes	Yes	Yes	Yes	Yes	–	CHIKV	No
4	<18/F	April 23	2	14	Yes	No	Yes	No	Yes	Yes	Yes	Yes	No	–	CHIKV	No
5	>64/M	April 23	3	14	Yes	Yes	No	Yes	Yes	Yes	Yes	No	No	Diabetes mellitus	CHIKV/DENV	Dengue 1
6	18-24/F	April 23	3	15	Yes	No	No	Yes	Yes	Yes	No	No	No	–	CHIKV	No
7	18-24/M	April 23	4	14	Yes	No	No	Yes	Yes	Yes	No	No	No	–	CHIKV	No
8	35-44/M	April 23	3	21	Yes	No	No	Yes	Yes	Yes	No	No	Yes	–	CHIKV	No
9	35-44/F	April 23	5	21	Yes	No	Yes	Yes	Yes	Yes	Yes	No	No	Hypertension	CHIKV	No
10	<18/M	April 23	2	24	Yes	Yes	Yes	Yes	Yes	Yes	Yes	No	No	–	CHIKV	No
11	25-34/F	April 23	3	21	Yes	No	No	Yes	Yes	Yes	No	Yes	No	Pregnant (7 months)	CHIKV	No
12	18-24/M	April 23	2	15	Yes	Yes	No	Yes	Yes	No	No	No	No	–	CHIKV/DENV	Dengue 2
13	<18/M	April 23	3	14	Yes	Yes	Yes	Yes	No	No	No	No	No	–	CHIKV	Hemorrhagic febrile syndrome (epistaxis)
14	35-44/F	May 23	2	15	Yes	Yes	Yes	Yes	Yes	Yes	Yes	No	Yes	–	CHIKV/DENV	Dengue
15	18-24/F	May 23	2	16	Yes	Yes	No	Yes	Yes	Yes	Yes	No	Yes	–	CHIKV	No
16	25-34/M	March 23	2	N/S	Yes	Yes	Yes	Yes	Yes	Yes	Yes	No	No	–	CHIKV	No
17	18-24/F	March 23	3	N/S	Yes	Yes	No	Yes	Yes	Yes	No	No	No	–	CHIKV	No
18	25-34/F	March 23	2	N/S	Yes	Yes	No	Yes	Yes	Yes	Yes	No	No	–	CHIKV	No
19	35-44/M	April 23	4	N/S	Yes	No	No	Yes	Yes	Yes	Yes	Yes	No	–	CHIKV	No
20	35-44/M	April 23	2	N/S	Yes	Yes	Yes	Yes	Yes	Yes	Yes	No	No	–	CHIKV/DENV	Dengue 2

A = acute; CHIKV = chikungunya virus; DENV = dengue virus; F = female; M = male; N/S = no sample available.

To investigate whether CHIKV induces cross-neutralizing immunity against MAYV, we selected the 15 paired samples available and carried out plaque reduction neutralization tests with the MAYV strain FPI00179 (genotype D) and the CHIKV vaccine strain 181/25 obtained from the World Reference Center for Emerging Viruses and Arboviruses at the University of Texas Medical Branch at Galveston. A MAYV genotype D strain was chosen because of its broad geographic distribution across Latin America and the Caribbean, making it the most widespread of the three known MAYV genotypes (D, L, and N). Additionally, using genotype D ensured consistency with previous studies that had evaluated cross-protection between CHIKV and MAYV after natural human infection.^13^^,^^15^^,^^22^ Virus stocks were prepared in Vero cells (CCL-81, ATCC, Manasas, Virginia), aliquoted, and titrated as previously described.^9^ Sera samples were diluted 1:10 and heat inactivated through incubation at 56°C for 1 hour. All acute and convalescent samples were assayed by performing plaque reduction neutralization tests as published previously.^22^ The 80% plaque reduction neutralization titer (PRNT_80_) for each sample was determined as the highest serum dilution that reduced plaque formation by >80%. Titers less than 20 were considered to be negative for neutralizing antibodies against the respective virus.

## RESULTS

One hundred percent (*n* = 15/15) of the convalescent CHIKV-immune sera samples had neutralizing antibodies against CHIKV, with PRNT_80_ values ranging from 80 to ≥640 (Table 2). About 47% of the convalescent CHIKV samples (*n* = 7/15) showed cross-neutralization to MAYV *in vitro*. However, it was still necessary to rule out if these patients might have had previous MAYV exposure.

**Table 2:** Chikungunya virus- and Mayaro virus-neutralizing antibody levels in acute and convalescent sera from chikungunya virus-infected patients in Mariscal Estigarribia, Paraguay in 2023

Patient	Acute (PRNT_80_)	Convalescent (PRNT_80_)	
	MAYV	CHIKV	MAYV
1	<20	160	<20
**2**	**<20**	**≥640**	**40**
3	80	≥640	80
4	<20	320	<20
**5**	**<20**	**80**	**20**
6	<20	80	<20
7	<20	≥640	<20
8	640	≥640	640
9	<20	≥640	<20
10	<20	320	<20
11	<20	320	<20
12	20	≥640	20
**13**	**<20**	**160**	**20**
14	<20	320	<20
**15**	**<20**	**320**	**20**

Bolded values denote CHIKV-infected patients with MAYV-neutralizing antibodies present in convalescent sera, who had no evidence of prior immunity to MAYV.

CHIKV = chikungunya virus; MAYV = Mayaro virus; PRNT_80_ = 80% plaque reduction neutralization titer.

Even though the circulation of MAYV had not been reported in Paraguay, we next investigated whether the MAYV neutralization-positive participants had pre-existing MAYV immunity before CHIKV infection. This was accomplished by evaluating the presence of MAYV-neutralizing antibodies on the acute-phase sera samples that were positive for CHIKV viral RNA. We found that three of these seven MAYV neutralization-positive patients indeed had the same level of neutralizing antibodies in their acute-phase sera samples, indicating that they were, in fact, exposed to MAYV before CHIKV infection. Although three individuals showed serological evidence of prior MAYV exposure, this observation arose from our neutralization assays and was not an original aim of the study. Notably, none of these patients had evidence of anamnestic responses because the MAYV cross-neutralization titers were the same in both acute-phase and convalescent-phase samples (Table 2). Thus, of the 12 other CHIKV patients without previous MAYV exposure, approximately 33% developed relatively low MAYV cross-neutralizing antibody responses, with titers ranging from 20 to 40. Their convalescent PRNT_80_ values for MAYV were all lower by between 4-fold and 16-fold than for the respective neutralization titers for CHIKV.

## DISCUSSION

This study found that CHIKV infection generated low levels of cross-neutralizing antibodies against MAYV among one third of the patients without prior MAYV exposure. Our results differ from other human studies that reported a higher percentage of MAYV cross-reactivity and titer levels after natural CHIKV infection or vaccination.^4^^,^^13^^,^^16^^,^^19^ Several factors could contribute to these contrasting results. First, previous studies did not definitely verify prior MAYV immunity among their patient populations. Second, it is possible that differences between antibodies induced by natural versus vaccine-induced CHIKV infection exist. However, in contrast to past human studies, we were able to definitively assess prior MAYV immunity among CHIKV-infected patients by using paired sera samples from the acute and convalescent phases. Third, potential differences in the circulating CHIKV and MAYV genotypes in Paraguay might also have influenced these results, a possibility that warrants further investigation. Despite these considerations, our results conclusively ruled out past MAYV exposure and consistently demonstrated only limited levels of MAYV cross-reactivity.

Additionally, we detected previous exposure to MAYV in 3 of 15 patients who developed febrile illness after CHIKV infection, suggesting that pre-existing MAYV immunity may not be sufficient to protect against secondary CHIKV infection. This further emphasizes the importance of ruling out prior infection in future cross-neutralization studies. These results are consistent with our previous report on MAYV-infected patients.^22^ Taken together, our results suggest that CHIKV may generate limited amounts of cross-neutralizing antibodies against MAYV *in vitro* but not the other way around.^22^ This finding suggests a non-reciprocal pattern in cross-protection between MAYV and CHIKV, a pattern that could potentially extend to other alphaviruses and should be further investigated. Although MAYV and CHIKV share high sequence similarity in domain A of the E2 glycoprotein, these similarities did not translate to broad cross-protective immunity or high levels of cross-reactive neutralizing antibodies. Several factors can explain these discrepancies, including that even minor amino acid differences in specific neutralizing epitopes can alter antibody binding affinity and specificity. Additionally, the immune response might be primarily directed toward immunodominant epitopes that are unique or highly divergent between these two viruses. Despite their close evolutionary relationship and shared structural features, our results suggest that the specific epitopes critical for inducing protective immunity against CHIKV and MAYV are sufficiently distinct to limit significant cross-protection.

In light of these results, there is a renewed need for a safe and effective vaccine that specifically protects against MAYV infection. As we did not find strong evidence of cross-reactive responses between CHIKV and MAYV, there is greater value in a future vaccine strategy in MAYV-endemic regions that targets MAYV specifically rather than relying on CHIKV vaccine-induced immunity. Because of the increased frequency and increased geographic expansion of recent MAYV outbreaks, global preparedness efforts should be proactively directed to disease countermeasures, such as MAYV vaccine development.^2^ Furthermore, it is also necessary to determine to what extent cross-protection potency correlates with protection of a disease, which is not fully clear and merits further study.

The study also found evidence of a potential silent circulation of MAYV in Paraguay, which requires further investigation. Given the similar clinical presentations in MAYV- and CHIKV-infected patients, it is possible that MAYV infections are being misdiagnosed as CHIKV, and studies are needed to evaluate the full extent of human exposure to MAYV in Paraguay. These findings have important implications on the civilian population, deployed military personnel, and operational units in MAYV and CHIKV co-endemic areas. They also contribute to a better understanding of the natural cross-reactive immunological responses among alphaviruses.

## CONCLUSION

The limitations of this study primarily relate to the limited size of the CHIKV-infected patient cohort as well as the fact that all of the sera samples were from Paraguay. Additional serological research is needed to evaluate the extent of endemic MAYV circulation in Paraguay, but our study provides preliminary evidence suggesting that additional research efforts should be directed to these investigations. We also acknowledge the possibility that the three individuals with MAYV positivity might have been exposed to the virus in a different location. This detail could not be captured by our questionnaire, which was limited to trips within the last 15 days before symptom onset. Nevertheless, it is notable that at least one individual reported never having traveled outside Paraguay, suggesting that MAYV exposure might have occurred within the country. Looking ahead, we also acknowledge the value of larger human cohort studies in the future that assess cross-protective responses between CHIKV and MAYV and can more extensively examine the conclusions of this study.
